# Effect of Void Defects on the Indentation Behavior of Ni/Ni3Al Crystal

**DOI:** 10.3390/nano13131969

**Published:** 2023-06-28

**Authors:** Longlong Yang, Kun Sun, Huaying Wu

**Affiliations:** 1 State Key Laboratory for Mechanical Behavior of Materials, Xi’an Jiaotong University, Xi’an 710049, China; yanglonglong@stu.xjtu.edu.cn; 2 School of Mechanical Engineering, Xi’an Jiaotong University, Xi’an 710049, China; wuhy@xjtu.edu.cn

**Keywords:** MD simulation, indentation, Ni/Ni3Al crystal, void, mechanical properties, dislocation

## Abstract

Inconel 718 (IN 718) superalloys are widely used as engineering materials owing to their superior mechanical performance. And voids are unavoidable defects in IN 718 superalloy preparation, which dramatically affect the mechanical properties of IN 718 superalloys. In this work, the effects of void radius, distance from the top of the void to the substrate surface, and substrate temperature on the mechanical properties of the Ni/Ni3Al crystal are systematically investigated. It is shown that voids affect the formation of stair-rod dislocations and Shockley dislocations in the substrate, which in turn determines the mechanical properties. Thus, with the increase in void radius, Young’s modulus and hardness gradually decrease. With the increase in void distance, Young’s modulus and hardness increase and finally tend to be stable. In addition, the increase in substrate temperature leads to the interphase boundary becoming irregular and increases the defects in the γ and γ″ phases. As a result, Young’s modulus and hardness of the substrate decrease. This work aims to provide a guideline for investigating the indentation properties of Ni-based superalloys using MD.

## 1. Introduction

Inconel 718 (IN 718) is a typical precipitation-strengthened nickel-based superalloy that has been widely used in hot section parts of the nuclear energy industry, and in aero-engine and petroleum applications owing to its superior mechanical properties at high-service-temperature conditions [1,2,3]. It is derived mainly from the FCC (face-centered cubic) γ-Ni matrix, carbides, stable phase δ (Ni3Nb), strengthening phase γ′ (Ni3(Al, Ti, Nb)), and γ″ (Ni3Nb) [2,4,5]. The long-range ordered L1_2_ intermetallic γ′-Ni3Nb and D0_22_ intermetallic γ″-Ni3Nb are believed to be efficient obstacles to the glide of dislocation in the Ni matrix [6,7,8]. Therefore, the investigation of the microstructure and mechanical properties of γ′-Ni3Nb and γ″-Ni3Nb is conducive to intrinsically understanding the mechanism of the excellent fundamental mechanics of IN 718 superalloys.

Over the past several decades, numerous experimental tests have been carried out to investigate the mechanical properties and deformation mechanisms of Ni-based superalloys. Research papers related to various aspects of Ni-based superalloys were reviewed by Selvaraj et al. [9]. There are four major categories of Ni-based superalloys: manufacturing, effects of alloy elements, mechanical properties, and defects in Ni-based superalloys. This review provides insight into the achievements of Ni-based superalloys. Regarding the manufacturing of IN 718 superalloys, Du et al. [2] summarized the advance of IN 718 superalloys, including alloy modification and hot deformation technology. And Zhang et al. [10] investigated the effects of laser power and scanning speed of selective laser melting technology on the density of IN 718 superalloys. Alloying elements play a decisive role in the mechanical properties of Ni-based superalloys; therefore, Long, Das, Huang, and Hata et al. [1,11,12,13] studied the effect of alloying elements on the microstructure and properties of Ni-based superalloys, and divided the alloying elements into four categories: base elements, strengthening elements, stability elements, and oxidation resistance elements. The geometrical stress concentrations created by microstructure defects, like microvoids and microcracks, play a significant role in the localization of the structural response of Ni-based superalloys [14]. Liu et al. [15] observed that microvoids are often formed at the intersection of two sets of slipping dislocations. Yu et al. [16] found that microvoids caused a local stress concentration for crack propagation, finally resulting in the fast fracture.

On the other hand, thanks to the rapid improvement of computer technology in recent decades, atomic simulation methods have been widely used to explore the performance of Ni-based superalloys, for instance, first-principles, molecular dynamics (MD), Monte Carlo (MC), phase field crystal (PFC), and machine learning [17,18,19,20,21]. In these methods, MD simulation can provide an accurate representation of the demonstration of material deformation at the nanoscale. Li et al. [19] studied the effect of various temperatures and strain rates on the evolution of dislocation and mechanical properties at the γ and γ′ phases’ interface. Khoei et al. [22] employed MD to investigate the creep deformation mechanisms at the various conditions of temperatures, stress, and phase interface orientations and predicted the steady-state strain rate. Zhou et al. [23] used MD to simulate nanoindentation and explore the evolution of the microstructure at different indent locations of Ni-based single-crystal alloy.

Void defects were unavoidably introduced during IN 718 superalloys’ preparation, which not only affected the mechanical properties but also changed the deformation mechanism. Both experiment and simulation have been employed to reveal the essence of the effect of voids on the mechanical properties of IN 718. Wang et al. [24] reported that the dislocations initially nucleate on the surface of voids and the mechanical properties are controlled by the interaction between void and interface. Shang et al. [25] discovered that the interaction between void and interface triggers varying dislocation nucleation and plastic deformation patterns. Cui et al. [26] found that the slipped dislocation can swimmingly enter into the γ′ phase along the periphery of the void to cause the reduction in mechanical strength. Based on these studies of the effect of voids on the mechanical properties, in this work, MD is employed to explore the incipient plasticity in γ/γ′-Ni/Ni_3_Al interface indentation. Additionally, considering the size and location effect of pre-existing voids in the model, there are various models with different void radii and distances from voids to substrate surface to evaluate the incipient plasticity of the γ/γ′ system. This work aims to better understand the deformation mechanisms and the weakening mechanics of voids in a γ/γ′ system.

## 2. Calculation Methods

**Figure 1:**
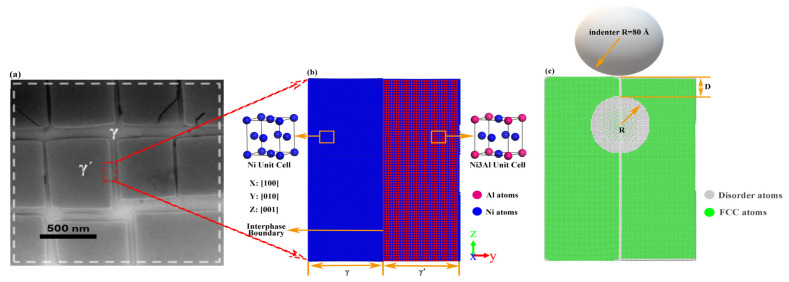
Atomic configuration diagram of the γ/γ′ system model. (**a**) TEM [34]; (**b**) initial model; (**c**) indent model.

As previous studies have shown, it is well known that there is a mismatch of δ crystal lattices of γ and γ′ phases, and δ is described as [27]

(1) $$\delta =\frac{2(\alpha_{\gamma^{\prime }}-\alpha_{\gamma })}{\alpha_{\gamma^{\prime }}+\alpha_{\gamma }}$$

where $\alpha_{\gamma^{\prime }}$ and $\alpha_{\gamma }$ are the crystal lattice parameters of Ni_3_Al and Ni, respectively. According to the empirical data [27,28], $\alpha_{\gamma^{\prime }}$ and $\alpha_{\gamma }$ are 3.52 Å and 3.571 Å, respectively. Furthermore, the misfit stress is alleviated by constructing a mismatched phase interface, and the misfit is described as

(2) $$n\alpha_{\gamma^{\prime }}=\left(n+1\right)\alpha_{\gamma }$$

where n is the number of unit cells. In Equation (1), the unknown variable of n is 66. As shown in Figure 1a, the initial model is established by γ and γ′ phase stacking along the Y-direction [010], the γ phase block is 67 × 30 × 67 $\alpha_{\gamma }$, and the γ′ phase block is 66 × 30 × 66 $\alpha_{\gamma \prime }$. To establish the initial models, the Swiss-army knife of atomic simulation (ATOMSK) [29] is employed to merge the γ phase and γ′ phase together along the *Y*-axis. Furthermore, large numbers of experiments and theoretical analysis show that the γ-Ni and γ′-Ni3Al usually have an interface dependency along the direction of (001)γ || (001) γ′ [30,31]. The γ phase and γ′ phase include 538,680 atoms and 522,720 atoms, respectively. Based on this initial model, a series of voids with different radii are set in different locations, as shown in Figure 1b. According to the study of the indenter radius on the influence of system characteristics, the indenter radii should be selected as larger than 60 Å [32]. Thus, in this work, the radius of the indenter is selected at 80 Å to investigate the mechanical properties of the γ/γ′ system. A force between the spherical indenter and substrate can be described as

(3) $${F(r)=-K(r-R)}^{2}$$

where K is the specified force constant, equal to 10 $eV/{Å}^{3}$ [32,33]. R and r are the radius of the indenter and the distance between the atom and the center of the indenter, respectively. Moreover, it is worth mentioning that the force F(r) is repulsive. By adopting a virtual indenter to explore the indentation properties of the substrate, the substrate atoms can be effectively prevented from jumping out of the substrate and adhering to the indenter. The different locations and radii of the void significantly affect the dislocation–precipitate interactions in the Ni-based superalloys substrate [26]. Thus, as shown in Figure 1b, a void of different locations and radii is prepositioned at the interphase boundary. The different locations of the voids are mainly reflected in the distance from the top of the void to the substrate surface. In this work, the radius *R* and distance *D* of voids all vary from 0 to 60 Å.

The indentation simulations are carried out in the Large-scale Atomic/Molecular Massively Parallel Simulator (LAMMPS) package [35]. During indentation, periodic boundary conditions are introduced in the X and Y direction, while non-periodic and shrink-wrapped boundary conditions with a minimum value are implemented along the Z direction. The embedded-atom method (EAM) potential function developed by Mishin et al. [28] is selected to define the atomic interactions between the present γ/γ′ system substrate atoms, which is an EAM potential that was developed for the Ni3Al system in 2004. This potential has been used to study the interphase boundary structure and atoms sliding for the Ni-Al system [23,36,37].

The energy minimization method is employed to maintain the stability of the γ/γ′ system and the Nose–Hoover thermostat [38] is employed to control the temperature. With this configuration, at a γ/γ′ system pressure of 0 bar and temperatures of 0.1 K, 300 K, 600 K, 900 K, 1000 K, and 1100 K, every model is equilibrated for 75 ps in an isobaric–isothermal ensemble (NPT) [39]. In addition, in the simulation of exploring the effect of void location and radii, MD simulations are carried out at 0.1 K to avoid the effect of thermal fluctuation on dislocation processes. The sphere indenter pushes into the substrate at a constant velocity of 0.25 Å/ps. To avoid the substrate moving in the process of the sphere indenter pushing into the substrate, 10 layers of atoms are fixed at the bottom of the substrate. Thus, the depth *h* of the indenter can be obtained from the position of the tip of the spherical indenter tip. The velocity Verlet integrator with a time step of 1 fs is employed for all simulations. The indentation resulting structure is visualized by using the Open Visualization Tool (OVITO) [40]. The atomic configuration and dislocation evolutions are analyzed by the Dislocation Extraction Algorithm (DXA), which identifies all dislocations in the substrate, determines Burgers vectors of dislocation, and outputs lines with different colors representing different types of dislocations [40,41].

## 3. Results and Discussions

### 3.1. Influence of the Void Radius

**Figure 2:**
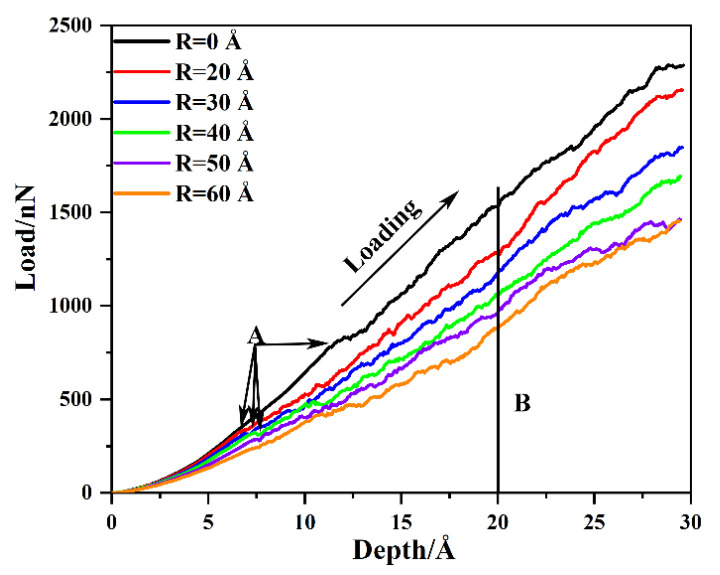
The load–depth curves of the γ/γ′ system model with various void radii. Point A represents the yield depth and point B represents the point at an indentation depth of 20 Å.

It is well known that the radius of voids severely weakens the mechanical properties of the substrate material. Thus, in this work, the mechanical properties and dislocation evolution mechanism of the preset void model with various radii are investigated under the condition that the distance from the top of the void to the substrate surface is *D* = 20 Å. And the load–depth curves of the γ/γ′ system model with various void radii *R* are shown in Figure 2. The load–depth curve of the γ/γ′ system model with various void radii can be divided into two stages, the elastic stage and plastic stage. It can be found that with the increase in the void radius, the mechanical properties of the γ/γ′ system model are significantly reduced, including not only Young’s modulus and hardness in the elastic stage but also the flow stress in the plastic stage. This result is consistent with Cui et al. [26], finding that dislocations can enter into the γ′ phase along the periphery of the void, which leads to the decrease in the resistance of the γ′ phase dislocation to glide smoothly, and then leads to the decrease in mechanical strength.

To study the effect of the void radius on the elastic response of the γ/γ′ system model, the elastic parameters of all models are illustrated, and the results are shown in Table 1. Firstly, regarding the pop-in at the load–depth curve, as shown in Figure 2—point A and Table 1, the depth and load of the pop-in first increase and then decrease as the void radius increases, regardless of the void-free model. When the void radius *R* = 40 Å, point A has the largest penetration depth and load, which are 7.5 Å and 321.39 nN, respectively. According to the Hertzian contact model [42], Young’s modulus can be fitted to penetration depth and load before the first load drop. And the relation between the applied load *P* and penetration depth *h* can be described as

(4) $$P=\frac{4}{3}E_{r}*R^{1/2}*h^{3/2}$$

where $E_{r}$ is the indentation modulus, and all the fitted values of $E_{r}$ are shown in Table 1.

The Young’s modulus of the indentation induced in the Ni3Al crystal at the [100] crystal orientation is 191 GPa [43]. And Young’s modulus for the R = 0 Å of γ/γ′ system model is 168.39 GPa; considering the γ phase and interphase boundary, the fitted values are valid. As shown in Table 1, the results indicate that the preset void in the substrate can severely weaken the fitted Young’s modulus, and Young’s modulus values decrease gradually with the void radius increase. Especially when the void radius is larger than 40 Å, Young’s modulus values drop sharply. The weakening of Young’s modulus by the void in the substrate is due to the fact that Young’s modulus completely depends on the integrity of the atomic structure below the indenter [44]. The preset void destroys the integrity of the atomic structure and increases the number of defective atoms below the indenter, so the fitted Young’s modulus decreases with the void radius increase. And according to Hertzian theory, the contact pressure is the substrate response to the indentation in the normal direction. Thus, the fitted hardness can be described as

(5) $$H=\frac{P}{S}=\frac{P}{\pi h(2R-h)}$$

Using Equation (5), the fitted hardness values of the γ/γ′ system model are shown in Table 1. Compared with the model with void *R* = 0 Å, the fitted hardness values decrease by 31.23%, 35.30%, 38.42%, 45.82%, and 53.70%, respectively. This result indicates that the void weakens the hardness of the substrate, and the weakening is more serious with the increase in the void radius.

**Figure 3:**
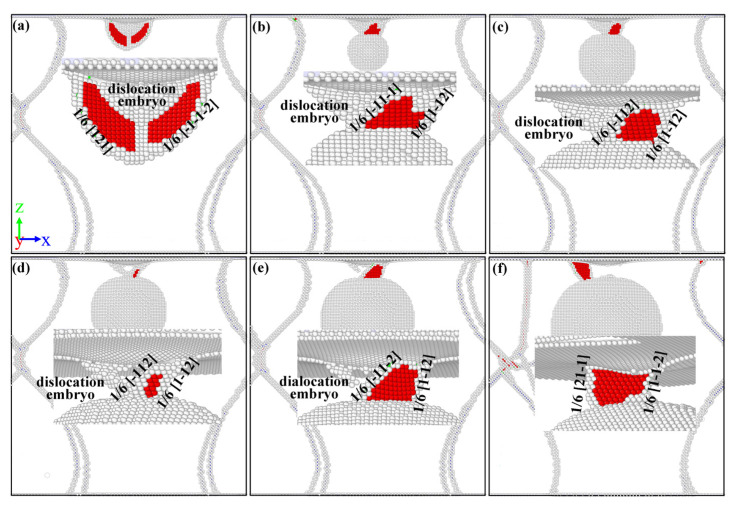
The dislocation nucleation of (**a**) *R* = 0 Å, (**b**) *R* = 20 Å, (**c**) *R* = 30 Å, (**d**) *R* = 40 Å, (**e**) *R* = 50 Å, (**f**) R = 60 Å view from the front. Gray atoms are disorder atoms and red atoms are the stacking fault atoms.

To study the effect of void radius on dislocation nucleation, the atomic configuration diagrams of different model are analyzed and shown in Figure 3. For legible visualization, the FCC structure atoms have been removed. Generally, the dislocation nucleation of the γ/γ′ system model forms a dislocation embryo and Shockley partial dislocation below the substrate surface. In the model without a void, as shown in Figure 3a, a dislocation embryo that aggregates into a γ phase and Shockley partial dislocation that extends into a γ′ phase is clearly observed. This result is consistent with the results by Cui et al. [26] where dislocation loops will enter the precipitate (γ′) phase with the increase in strain. As the void radius increases, the dislocation embryo gradually becomes smaller or even disappears, as shown in Figure 3b–f. After the plastic deformation takes place, the initial dislocation loops will expand on the {111} slip planes and intersect with other Shockley partial dislocation loops on the adjacent {111} planes as well, where two Shockley partial dislocations form a “butterfly-like” junction structure as shown in Figure 3a. The interstitial prismatic loops are generated in the substrate while the “butterfly-like” is formed. In addition, compared to the initial dislocation in Figure 3, a difference in the initial dislocation shape can be observed. In the void-free model, the dislocation shape is “butterfly-like”, and in the mode with a void, the dislocation shape is an interstitial prismatic loop. This indicates that the presence of a void in the γ/γ′ system model directly affects the shape of the initial dislocation and will have an effect on the subsequent dislocation expansion as well. Therefore, the internal dislocation of different models at different penetration depths is analyzed.

**Figure 4:**
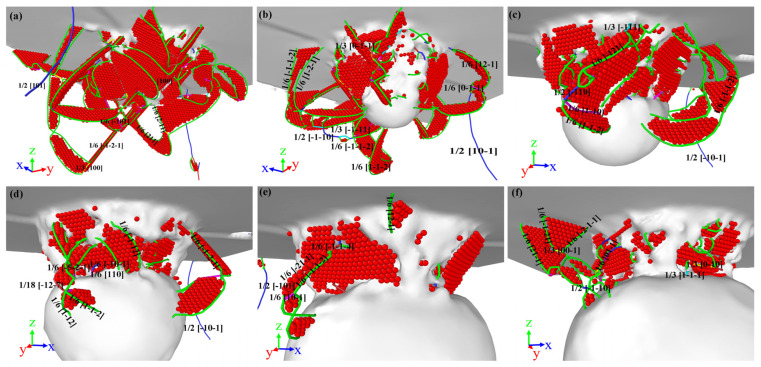
Snapshots for the dislocation loops at penetration depth *h* = 20 Å. (**a**) *R* = 0 Å, (**b**) *R* = 20 Å, (**c**) *R* = 30 Å, (**d**) *R* = 40 Å, (**e**) *R* = 50 Å, (**f**) *R* = 60 Å. Red atoms represent the stacking fault atoms, green lines are Shockley partial dislocation lines, pink lines are the stair-rod dislocations, yellow lines are Hirth dislocations, and blue lines are perfect dislocations.

In consideration of the distance from the top of the void to the substrate surface *D* = 20 Å, the atomic configuration at penetration depth *h* = 20 Å is analyzed and the results are shown in Figure 4. With the rise in penetration depth, dislocation loops appear in the substrate, slipping along the direction of <101> [45,46]. Compared with Figure 3, there are more “butterfly-like” junction structures and interstitial prismatic loops in Figure 4. Both of these structures play a significant role in plastic deformation. With the rise in penetration depth, more dislocation loops are emitted along the directions of [01$\overset{-}{1}$] and [10$\overset{-}{1}$] [46]. Then, dislocations expand and multiply deep inside the γ/γ′ substrate, leading to load fluctuation, but the overall trend is increasing as shown in Figure 2.

Compared with Figure 4a–f, as the radius of the void in the substrate increases, the dislocation expansion is concentrated between the void and the surface of the substrate. Even if there are dislocations that extend to the interior of the substrate, they will eventually slip to the surface of the void as shown in Figure 4c,d,f. When the γ/γ′ substrates contain a preset void, the preset void becomes a new dislocation source, and the Shockley partial dislocation around the preset void will not successfully enter into the substrate [26]. The interaction of the dislocation plays an important role in the plastic deformation stage, such as a dislocation kink, dislocation tangle, dislocation jog, and pile-up of dislocations. At penetration depth *h* = 20 Å, the stair-rod dislocation (1/6 <110>, pink line), Hirth dislocation (1/3 <001>, yellow line), perfect dislocation (1/2 <110>, blue line), and other dislocations (red line) are produced in the substrate. As shown in Figure 4, two Shockley dislocations react to form the stair-rod dislocation and Hirth dislocation [47], and this reaction can be described as

(6) $$1/6\left[1\overset{-}{1}2\right]\cdot \left(111\right)+1/6[1\overset{-}{1}\overset{-}{2}]\cdot (111)\to 1/6[1\overset{-}{1}0]$$

(7) $$1/6[2\overset{-}{1}1]+1/6[\overset{-}{2}11]\to 1/3[001]$$

According to the analysis of DXA, when penetration depth *h* = 20 Å, the calculated dislocation density *ρ* in the substrate is $4.1\times 10^{-3}/{Å}^{-3}$, $2.6\times 10^{-3}/{Å}^{-3}$, $1.9\times 10^{-3}/{Å}^{-3}$, $1.5\times 10^{-3}/{Å}^{-3}$, $1.3\times 10^{-3}/{Å}^{-3}$, and $1.25\times 10^{-3}/{Å}^{-3}$. This indicates that the void, as a defect in the substrate, anchors the expansion of dislocation lines in the substrate and an abundance of dislocation lines pile up on the surface of the void. In addition, the surface of the void is a stress concentration area and alleviates the stress inside the substrate due to the indentation, thus weakening the power of dislocation propagation. Therefore, with the increase in void radius, the dislocation density *ρ* in the substrate decreases gradually.

**Figure 5:**
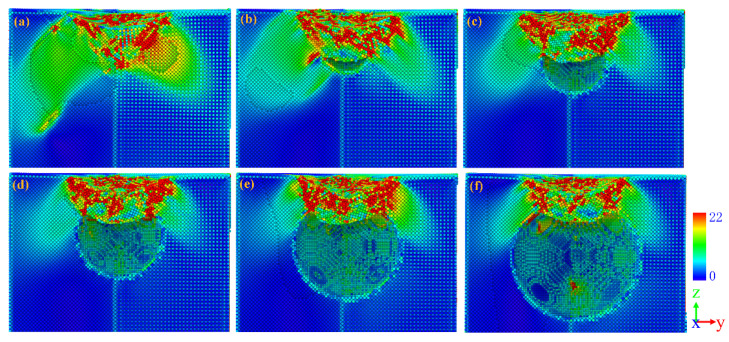
Cross-sectional view of von-Mises stresses at penetration depth *h* = 20 Å. (**a**) *R* = 0 Å, (**b**) *R* = 20 Å, (**c**) *R* = 30 Å, (**d**) *R* = 40 Å, (**e**) *R* = 50 Å, (**f**) *R* = 60 Å.

As concluded above, the void has a significant effect on the dislocation propagation in the substrate. To clearly understand this phenomenon, the von-Mises stress $\sigma_{\nu }$ is calculated by Equation (8):

(8) $$\sigma_{\nu }=\sqrt{\left[{\left(\sigma_{11}-\sigma_{22}\right)}^{2}+{\left(\sigma_{22}-\sigma_{33}\right)}^{2}+{\left(\sigma_{33}-\sigma_{11}\right)}^{2}+6{\left(\sigma_{12}^{2}+\sigma_{23}^{2}+\sigma_{31}^{2}\right)}^{2}\right]/2}$$

where $\sigma_{\mathrm{ij}}$ are calculated by the virial definition [48]. And the von-Mises stress at penetration depth *h* = 20 Å for all models is displayed in Figure 5. From the snapshots of von-Mises stress, it can be seen that von-Mises stress is concentrated between the void and the substrate surface with the increase in void radius. This explains the concentration of dislocation in this region in Figure 4.

### 3.2. Influence of the Void Distance

**Figure 6:**
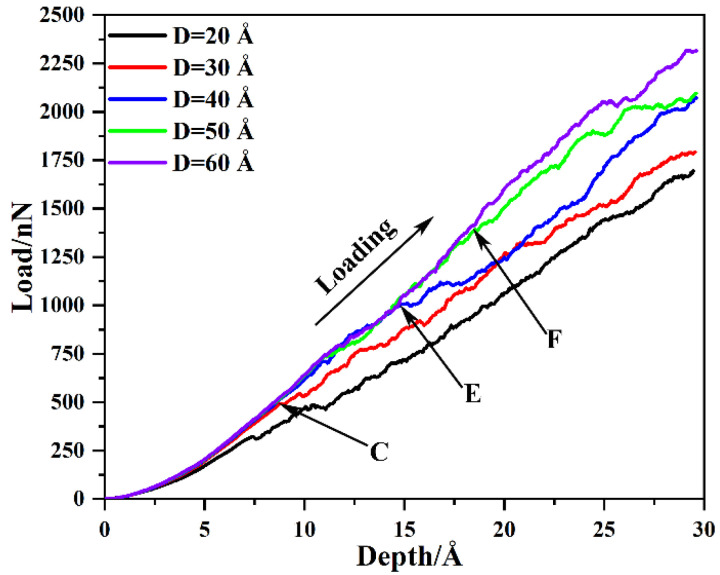
The load–depth curves of the γ/γ′ system model with various distances. The C, E and F points are the point at which the flow stress diverge increases.

In Section 3.1, the effect of the void radius on the indentation performance is discussed. Based on this, the effect of the distance *D* from the top of the void to the substrate surface with the void radius *R* = 40 Å is discussed in this chapter. And the load–depth curves of the γ/γ′ system model with various distances are shown in Figure 6. It can be seen from Figure 6 that the indentation properties of the substrate are significantly improved with the increase in void distance *D*, whether in the elastic or plastic deformation stages. Then, according to Equations (4) and (5), Young’s modulus and hardness are calculated for the different distances *D,* and the results are shown in Table 2. From Table 2, it can be seen that in the elastic deformation stage, depth, and load of pop-in, Young’s modulus and hardness increase with the increase in void distance *D*. However, when the void distance *D* increases to 40 Å, it has a slight effect on these properties. It can be seen from Figure 6 that in the plastic deformation stage, the flow stress of the substrate increases gradually with the increase in void distance *D*. Moreover, with the increase in void distance *D*, the penetration depth at which the load–depth curve begins to diverge increases, as shown at points C, E, and F in Figure 6. As mentioned above, the stress concentration area on the void surface directly affects the formation and expansion of dislocation rings in the matrix. Therefore, as the distance *D* increases, Young’s modulus and hardness of the matrix in the elastic deformation stage, as well as the flow stress in the plastic deformation stage, increase.

**Figure 7:**
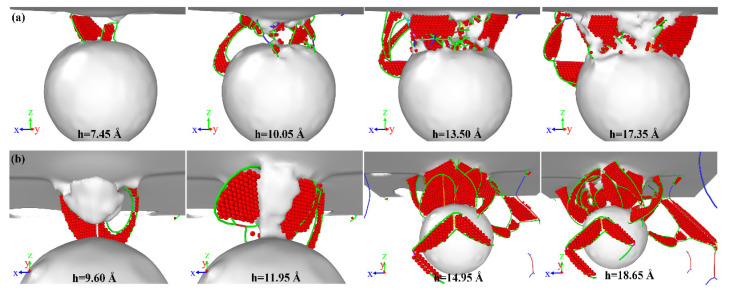
Variations in atomistic configuration under different penetration depths. (**a**) *D* = 20 Å; (**b**) *D* = 40 Å. The specification of line colors can be identified from the corresponding definitions in Figure 4.

Since the load–depth curves for various distances are similar to each other, only the dislocation evolution of distance *R* = 20 Å and *R* = 40 Å models are compared in the following. As shown in Figure 7, the void distance *D* affects the evolution of dislocation in the substrate. Firstly, as discussed in Section 3.1, the dislocation loops in the substrate will slip toward the surface of the void. However, comparing Figure 7a,b, it can be seen that in the substrate with void distance *D* = 40 Å, the dislocation loops between the void and the substrate surface are more inclined to form interstitial prismatic loops than “butterfly-like” junction structures. This phenomenon is similar to the distribution of dislocations in the substrate at penetration depth *h* = 20 Å in Figure 4. Secondly, as shown in Figure 7b, there is a tri-prismatic loop in the substrate, which is not present in Figure 7a. It indicates that various void distances *D* affect the evolution of dislocation in the substrate. Thirdly, a careful comparison of the atomistic configuration in Figure 7 shows that the length of dislocation lines in the γ′ phase with the void distance *D* increases. In addition, when void distance *D* = 40 Å, more interstitial prismatic loops are formed in the γ′ phase. This is because there is an energy competition between crossing the fixed dislocations in the γ′ phase and slipping in the γ phase [26]. These fixed dislocations, like stair-rod dislocations near the interphase boundary or in the γ′ phase, vastly impede the slip of Shockley partial dislocations toward the γ′ phase, which leads to the dislocations having to glide to the γ phase, as shown in Figure 4 and Figure 7a. It illustrates that fixed dislocations play a major role in impeding the glissile dislocation slipping. For the void distance *D* = 40 Å model, when the penetration depth is less than 11.95 Å, the fixed dislocations play a major role in the dislocation slip. However, when the penetration depth is more than 11.95 Å, the slip energy plays a major role, as shown in Figure 7b.

**Figure 8:**
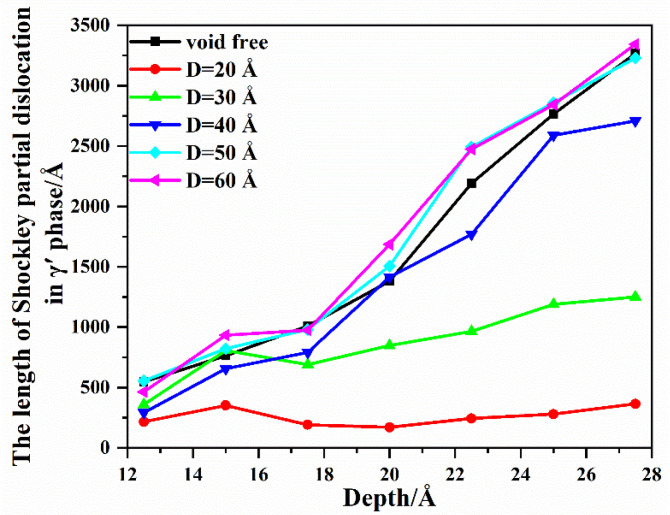
Shockley partial dislocation length in γ′ phase at various penetration depths.

To further prove the influence of the void distance *D* on the dislocations slipping in the γ′ phase, the DXA method is employed to calculate the length of the dislocations in the γ′ phase at various penetration depths. According to the atomistic configuration in Figure 7, the penetration depth changes from 12.5 Å to 27.5 Å (2.5 Å interval), and the length of the Shockley partial dislocation in the γ′ phase is shown in Figure 8. With the increase in penetration depth, for the model with void distance *D* = 20 Å, the length of the Shockley partial dislocation remains at a low level in the γ′ phase. For the model with void distance *D* = 30 Å, there is a certain increase in the length of the Shockley partial dislocation. For the model with void distance *D* = 40 Å, *D* = 50 Å, and *D* = 60 Å, the length of the Shockley partial dislocation increases significantly and is almost identical to that of the void-free model. This indicates that when the void distance *D* is larger than 40 Å, it has almost no effect on the slipping of Shockley partial dislocations in the γ′ phase.

### 3.3. Influence of the Void Temperature

**Figure 9:**
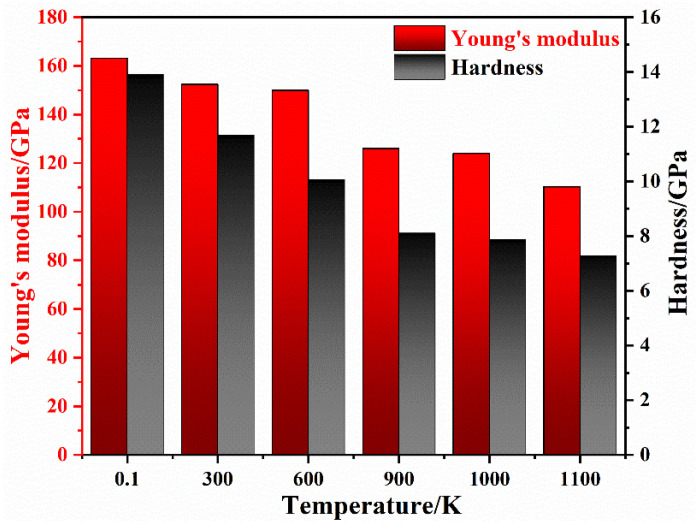
Young’s modulus and hardness at various temperatures.

Based on the exploration of void radius *R* and distance *D*, in this chapter, the preset void radius *R* = 40 Å and distance *D* = 40 Å of the γ/γ′ system model are employed to study the influence of temperature on indentation properties. And the MD-calculated Young’s modulus and hardness at various temperatures are shown in Figure 9. These Young’s moduli at various temperatures are 163.13 GPa, 152.40 GPa, 149.98 GPa, 126.06 GPa, 123.97 GPa, and 110.26 GPa. These hardness values at various temperatures are 13.91 GPa, 11.69 GPa, 10.07 GPa, 8.11 GPa, 7.86 GPa, and 7.27 GPa, respectively. As the temperature increases from 0.1 K to 1100 K, Young’s modulus reduces by 34% and the hardness reduces by 48%. When the temperature rises more than 500 K, the interphase boundary becomes unstable and generates damage [19], that is, before penetrating into the substrate, some region of the MD models below the indenter appear incomplete with a chaotic atomic structure. Thus, Young’s modulus and hardness decrease with the temperature increase. Moreover, it can be found that the model still maintains high Young’s modulus and hardness values at 900 K and 1000 K temperatures. This result is in agreement with the experimental results that Ni-based superalloys have excellent mechanical properties at high temperatures [22,44].

**Figure 10:**
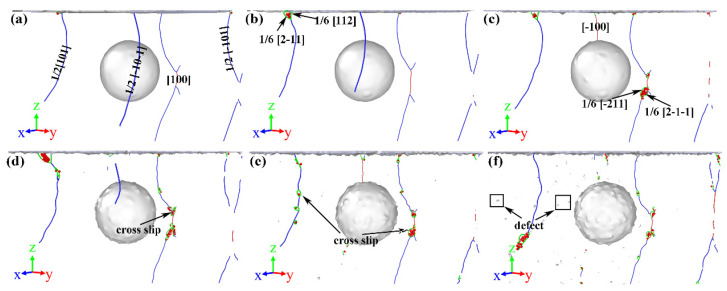
Morphologies of the dislocation lines in the γ/γ′ system model before indentation. (**a**) *T* = 0.1 K; (**b**) *T* = 300 K; (**c**) *T* = 600 K; (**d**) *T* = 900 K; (**e**) *T* = 1000 K; (**f**) *T* = 1100 K. The specification of line colors can be identified from the corresponding definitions in Figure 4.

To explore the effect of temperature on the indentation properties, the dislocations in the γ/γ′ system model at various temperatures before indentation are visualized and the results are shown in Figure 10. At *T* = 0.1 K, the dislocation type in the γ/γ′ system model is mainly perfect dislocation (1/2 <110>), as shown in Figure 10a. Shockley dislocations occur at the interphase and surface of the substrate and some perfect dislocation disappears with the increase in temperature, as shown in Figure 10b–e. And in Figure 10e,f, there are Shockley dislocation loops at the perfect dislocation line. It indicates that the interphase boundary becomes irregular and the irregular boundary is more likely to become damaged [19,27]. Then, the percent of disordered atoms in the γ/γ′ system model at various temperatures is 1.3%, 1.5%, 1.9%, 4.9%, 7.3%, and 10.8%. The percent of disordered atoms increases significantly. These disordered atoms are potential dislocation formation points. This explains the decrease in Young’s modulus and hardness in Figure 9 as the temperature increases from the atomic configuration.

## 4. Conclusions

In the present work, the indentation-induced plasticity of the γ/γ′ system model is investigated by employing MD simulation. The effects of void radius, void distance, and substrate temperature on the mechanical properties of the γ/γ′ system model are discussed in detail. The main conclusion can be summarized as follows:

(1) With the increase in the void radius, Young’s modulus, hardness, and flow stress decrease significantly. For dislocation nucleation, the larger the void radius is, the more conducive the Shockley dislocation and dislocation embryo are. For dislocation slip, voids act as a defect, which anchors the expansion of the dislocation in the substrate, effectively alleviating the stress.
(2) With the increase in the void distance, mechanical properties increase, and when the void distance *D* = 40 Å, the mechanical properties of all models are similar. The void distance affects the dislocation shape and type, and then affects the slip of the glissile dislocation in the γ′ phase.
(3) With the increase in the temperature, the mechanical properties decrease significantly, but still maintain a high level. The interphase boundary becomes irregular and the number of disordered atoms increase in the substrate. These defects increase the dislocation nucleation rate and leads to the decrease in mechanical properties.

## Figures and Tables

**Table 1:** MD-calculated elastic parameters of different void radii.

Parameter	Radius of Void/Å					
	0	20	30	40	50	60
Depth of the pop-in/Å	11.2	6.4	6.8	7.5	7.2	7.4
Load of the pop-in/nN	770.97	306.82	312.01	321.39	273.49	226.47
Young’s modulus/GPa	168.39	150.57	140.80	132.09	115.48	100.85
Hardness/GPa	14.73	10.13	9.53	9.07	7.98	6.82

**Table 2:** MD-calculated elastic parameters at different void distances.

Parameter	Distance of Void/Å				
	20	30	40	50	60
Depth of pop-in/Å	7.5	8.8	10.9	11.0	11.1
Load of pop-in/nN	321.39	489.56	711.44	736.80	740.31
Young’s modulus/GPa	132.09	155.84	163.13	166.95	167.96
Hardness/GPa	9.07	11.77	13.91	14.31	14.45

## Data Availability

The authors declare that all supporting the finding of this study are available within the article.
